# Systems Addressing Frail Elder Care: An Implementation Study

**DOI:** 10.1093/geroni/igaa057.854

**Published:** 2020-12-16

**Authors:** Harriet Aronow, Linda Burnes Bolton, Marcio Diniz, Linda Kim, Bernice Coleman

**Affiliations:** 1 Cedars Sinai, Los Angeles, California, United States; 2 Cedars Sinai Health System, Los Angeles, California, United States; 3 Cedars-Sinai, Los Angeles, California, United States

## Abstract

SAFE CareTM was developed at one hospital and found to be an effective care model for frail older adults. SAFE CareTM includes nurse screening for frailty risks, multidisciplinary assessments, team huddles and care recommendations. Underlying implementation is an organizational change process. Study aim was to evaluate the implementation and outcomes of SAFE CareTM in three additional hospitals. Two units from each hospital were randomized to SAFE CareTM or usual care. Process evaluation employed semi-structured interviews. Inpatients were aged 65+ years with positive frailty risks (N = 1,151). Outcomes evaluated ICU admission, length of stay (LOS), and discharge destination. All outcome analyses were conducted with intention to treat models. Patients were on average 80 years old, 54% female, 58% Caucasian, 83% English speaking, with 3.4 positive frailty risks. Median LOS was 4.2 days, 6.5% ICU admissions, 32% discharge institutional care. Hospitals differed in patient demographics and outcomes. While no differences between treatment groups in patient demographics, intervention patients had more frailty risks and longer expected LOS. 62% of intervention unit patients received intervention. There were no univariate treatment effects on outcomes. In multivariate analysis, intervention unit patients had shorter LOS. While hospitals reported different experiences, all reported challenges in preparing the electronic health record to support SAFE CareTM. Staff reported increased interprofessional team communications. Differences among the hospitals in patients and organizational attributes argue strongly that implementation should be tailored to meet varying institutional needs while common measures and processes underlying implementation should be followed closely.

